# The Rip1 intramembrane protease contributes to iron and zinc homeostasis in *Mycobacterium tuberculosis*

**DOI:** 10.1128/msphere.00389-22

**Published:** 2023-06-15

**Authors:** Samantha J. Nelson, John T. Williams, John A. Buglino, Subhalaxmi Nambi, Lisa J. Lojek, Michael S. Glickman, Thomas R. Ioerger, Christopher M. Sassetti

**Affiliations:** 1 Department of Microbiology and Physiological Systems, University of Massachusetts Chan Medical School, Worcester, Massachusetts, USA; 2 Immunology Program, Sloan Kettering Institute, New York City, New York, USA; 3 Department of Computer Science and Engineering, Texas A&M University, College Station, Texas, USA; University of Kentucky College of Medicine, Lexington, Kentucky, USA

**Keywords:** iron homeostasis, nutritional immunity, *Mycobacterium tuberculosis*, zinc, sigma factors

## Abstract

*Mycobacterium tuberculosis* is exposed to a variety of stresses during a chronic infection, as the immune system simultaneously produces bactericidal compounds and starves the pathogen of essential nutrients. The intramembrane protease, Rip1, plays an important role in the adaptation to these stresses, at least partially by the cleavage of membrane-bound transcriptional regulators. Although Rip1 is known to be critical for surviving copper intoxication and nitric oxide exposure, these stresses do not fully account for the regulatory protein’s essentiality during infection. In this work, we demonstrate that Rip1 is also necessary for growth in low-iron and low-zinc conditions, similar to those imposed by the immune system. Using a newly generated library of sigma factor mutants, we show that the known regulatory target of Rip1, SigL, shares this defect. Transcriptional profiling under iron-limiting conditions supported the coordinated activity of Rip1 and SigL and demonstrated that the loss of these proteins produces an exaggerated iron starvation response. These observations demonstrate that Rip1 coordinates several aspects of metal homeostasis and suggest that a Rip1- and SigL-dependent pathway is necessary to thrive in the iron-deficient environments encountered during infection.

**IMPORTANCE:**

Metal homeostasis represents a critical point of interaction between the mammalian immune system and potential pathogens. While the host attempts to intoxicate microbes with high concentrations of copper or starve the invader of iron and zinc, successful pathogens have acquired mechanisms to overcome these defenses. Our work identifies a regulatory pathway consisting of the Rip1 intramembrane protease and the sigma factor, SigL, that is essential for the important human pathogen, *Mycobacterium tuberculosis,* to grow in low-iron or low-zinc conditions such as those encountered during infection. In conjunction with Rip1’s known role in resisting copper toxicity, our work implicates this protein as a critical integration point that coordinates the multiple metal homeostatic systems required for this pathogen to survive in host tissue.

## INTRODUCTION

*Mycobacterium tuberculosis* (Mtb), the causative agent of tuberculosis, is one of the world’s most successful pathogens. Yearly, there are about 10 million newly diagnosed cases and more than 1 million deaths attributed to this disease (1). Part of this pathogen’s success lies in its ability to resist stresses caused by the host immune system, such as nutrient limitation or overload. Some metals, like copper and zinc, can be used to intoxicate pathogens in the phagolysosome (2, 3). Iron and zinc, in addition to other metals, are also sequestered by the immune system to starve the pathogen of these key micronutrients (2, 3). The ability to acquire iron either through scavenging host heme or binding scarce iron with siderophores is necessary for Mtb virulence (4 - 8). The possibility of oxidative stress from high levels of free iron and the high energy cost to produce and use siderophores means these systems are tightly regulated (9, 10). IdeR, the main iron regulator in Mtb, is an iron-binding transcription factor that represses transcription of iron acquisition genes when iron is high but dissociates from DNA when iron is low (6, 11). Similarly, Mtb has systems to adapt to either high or low concentrations of other metal ions such as zinc, copper, and manganese (12 - 20). The levels of each metal must be balanced to avoid loss of enzymatic activity due to oxidative stress, mismetallation, or the lack of a metallic cofactor.

To adapt to the changing environmental conditions encountered during infection, Mtb must first sense and then appropriately react with changes in gene expression or protein activity. This pathogen relies on an unusually complex network of sigma factors to tailor its transcriptional state to its environment. Sigma factors are an essential part of the RNA polymerase holoenzyme that recognizes the −10 and −35 regions of bacterial promoters. In Mtb, SigA is essential and considered the housekeeping sigma factor (21). The 12 other sigma factors, SigB-M, are dispensable *in vitro* and are activated in response to specific stimuli (22 - 36). Ten of these sigma factors belong to the family of extracytoplasmic function sigma factors that are thought to respond to extracellular stresses (37). The activities of several of these sigma factors are regulated by an anti-sigma factor that prevents RNA polymerase binding in the absence of stimuli (34, 38, 39). Some anti-sigma factors are membrane bound and are regulated by a proteolytic cascade. A site-1 protease proteolytically cleaves an anti-sigma, typically in response to a stimulus, priming it for further cleavage near the cytosolic side of the transmembrane domain by the site-2 protease, releasing the sigma–anti-sigma complex to the cytosol. The anti-sigma factor fragment is then degraded by cellular proteases, thereby releasing the sigma factor to bind RNA polymerase and allowing the cell to respond to the stress that was sensed (40, 41).

In Mtb, a single non-redundant site-1 protease has yet to be identified, but Rip1 (*Rv2869c*) has been extensively characterized as a site-2 protease that regulates SigD, SigK, SigL, SigM, and their regulons through proteolysis of their cognate anti-sigma factors (RsdA, RskA, RslA, and RsmA, respectively) (39, 42). Though only these four sigma factors are known to be controlled by Rip1, the sigma factor network in Mtb is highly interconnected. Coexpression of Mtb sigma factors in *Escherichia coli* demonstrated that most can regulate at least one but up to five others (43). Many of the sigma factors, including SigD, SigK, SigL, and SigM, directly upregulate their own expression as well as that of their anti-sigma factor (28, 36, 43 - 45). Due to the interconnected nature of this regulatory cascade and our lack of knowledge about the activating signals of each arm of the Rip1 pathway, it has proven difficult to delineate the direct and indirect effects of any upstream regulatory factor, such as Rip1, on downstream phenotypes.

While Rip1 is essential for Mtb replication in the mouse model of infection (42), the physiological roles for this regulatory system remain incompletely defined. Recently, it was shown that Rip1 integrates signals from nitric oxide and excess copper leading to expression of a small RNA that inhibits the PdtaR regulatory protein, allowing for expression of virulence-related genes and enhanced survival during infection (46). Deletion of iNOS in mice or deletion of *pdtaR* in a ∆*rip1* background partially rescued the ∆*rip1* fitness defect. These findings identified the first pathways regulated by Rip1 that directly contribute to its essentiality for infection. Still, the lack of full reversion of Rip1 attenuation by iNOS or *pdtaR* deletion suggests there are additional pathways or signals integrated by Rip1 beyond those described. As the gene expression of the iron storage protein, BfrB, was previously shown to be upregulated in *rip1*-deficient mutants compared with the wild-type strain, we explored the role of Rip1-dependent sigma factor cascades in iron homeostasis (39). We report that Rip1 plays an essential role in the adaptation to low-iron or low-zinc conditions, similar to stresses encountered during infection. Using a newly generated panel of mutants, we were able to systematically assess the contribution of each sigma factor to this phenotype, finding that the known target of Rip1, SigL, uniquely contributes to growth in low-iron media. Mutants lacking either Rip1 or SigL remain capable of inducing the canonical IdeR-dependent low-iron response suggesting that a novel mechanism related to iron acquisition or storage underlies this phenotype.

## RESULTS

### Rip1 is required for growth in low-iron conditions

**Fig 1:**
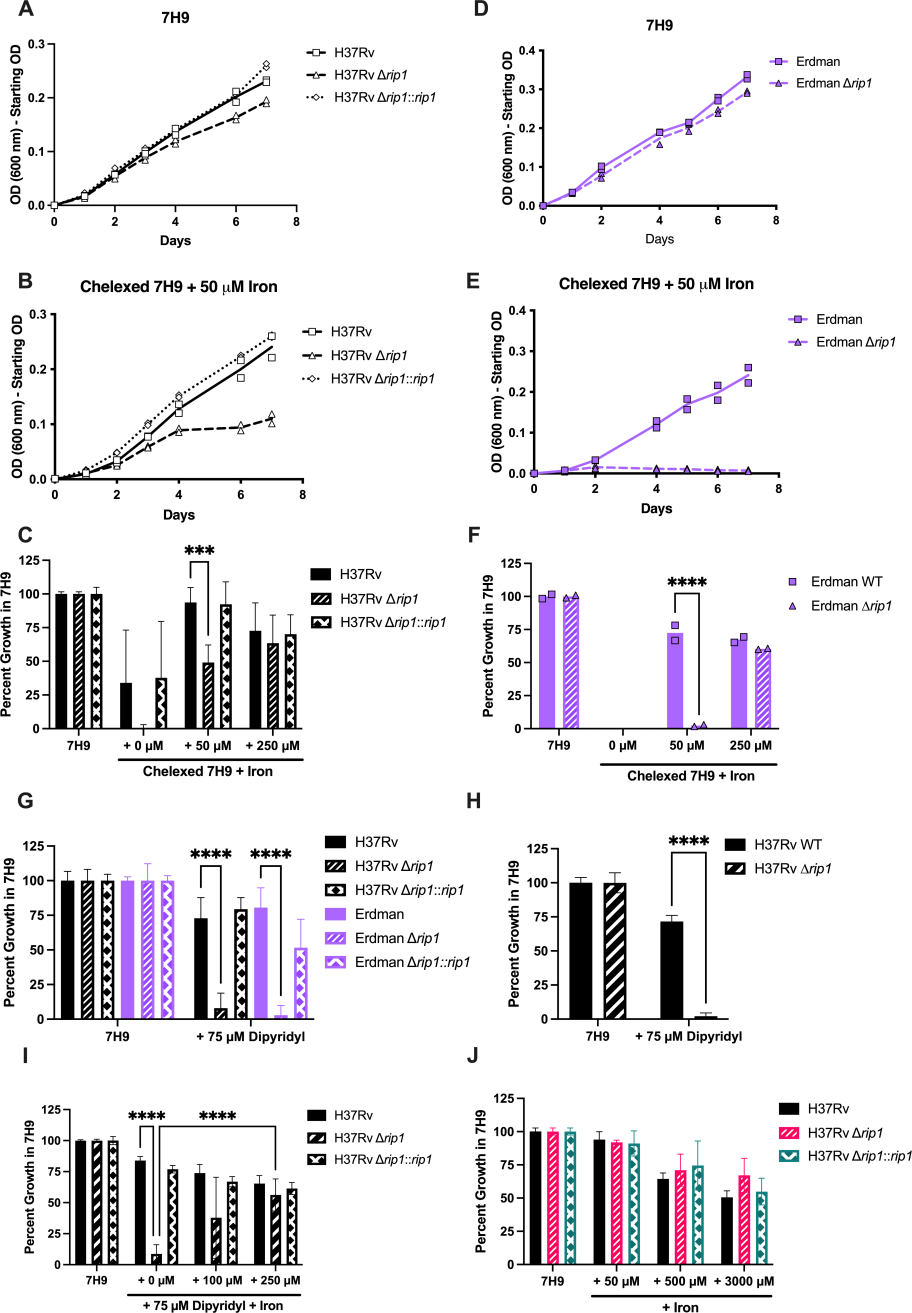
*rip1* is required for growth in low-iron conditions regardless of strain background. (**A–C**) Growth of the indicated strains in the H37Rv background in standard 7H9 (**A**) and chelexed 7H9 media with 50 µM ferric chloride (**B**) in 96-well plates. Representative mean and data for one out of three experiments shown. Normalized percent growth (**C**) calculated from (**A and B**) by dividing day 7 optical density (OD) in chelexed media by day 7 OD in standard 7H9 then multiplying by 100. Additional data shown for the condition with 0 µM and 250 µM added ferric chloride. Data depict mean and standard deviation for three experiments. (**D–F**) Same assays as described for (**A–C**), testing the indicated strains in the Erdman background. Representative plots shown for two experiments. (**G**) Percent growth calculated as described above for Erdman strains grown in standard 7H9 with and without 75 µM dipyridyl. Mean and standard deviation of five experiments shown. (**H**) Percent growth of indicated H37Rv strains in agitated broth cultures in standard 7H9 or with 75 µM of dipyridyl. Mean and standard deviation shown for combined data from two experiments completed in technical triplicate. (**I**) Percent growth of H37Rv strains in standard 7H9 compared with 7H9 with added dipyridyl and ferric chloride at noted concentrations. Data show mean and standard deviation of combined data from two experiments. (**J**) Percent growth of H37Rv strains in standard 7H9 with additional 50 µM, 500 µM, or 3,000 µM ferric chloride. Data show mean and standard deviation of combined data from two experiments. All experiments performed in technical duplicate in 96-well plates, incubated standing at 37° for 7 days unless otherwise stated. Growth measured by OD at 600 nm. Statistical analysis by two-way analysis of variance with Tukey’s multi-comparison correction. ****P < 0.0001; ***P < 0.001.

To test Rip1’s role in iron homeostasis, we first constructed a ∆*rip1* deletion mutant using ORBIT in *M. tuberculosis* H37Rv as well as a strain complementing the deletion mutant with *rip1* expressed under its native promoter (∆*rip1::rip1)* (47). To determine the effect of Rip1 on iron homeostasis, we made a low-iron media using chelex beads to remove divalent cations from 7H9, followed by replacing zinc, calcium, copper, and magnesium, and adding the stated amounts of ferric chloride. A representative growth curve shows that in standard 7H9 media, the ∆*rip1* mutant grew at a slightly slower rate than the wild-type (WT) parental strain (Fig. 1A). In contrast, growth of the ∆*rip1* strain was significantly inhibited in iron-depleted chelexed media (50 µM FeCl_3_), while the WT and complemented strains were unaffected (Fig. 1B). To account for the slightly slower growth of the mutant in ideal growth conditions (standard 7H9 media), we normalized the amount of growth in chelexed media to the total growth achieved for each strain in standard 7H9 (Fig. 1C). Chelexing successfully depleted iron from the media as demonstrated by the poor growth of the WT and complemented strains without iron addition. Achieving similar growth as in standard 7H9 required the addition of 50 µM of ferric chloride for the WT and the complemented mutant, while 250 µM of ferric chloride was required for the ∆*rip1* strain. To compare these studies to previous work on Rip1 in the *M. tuberculosis* Erdman strain and exclude any strain-specific phenotypes, we repeated these studies using a WT and a ∆*rip1* mutant in the Erdman background (39, 48). Consistent with our observations in the H37Rv background, the ∆*rip1* Erdman mutant was susceptible to iron depletion (Fig. 1D through F). Together, these observations show that the deletion of *rip1* impairs the ability of Mtb to grow in low-iron conditions.

As a complementary strategy to confirm that the phenotype of the ∆*rip1* mutant in chelexed media was due to iron limitation, we also tested the effect of dipyridyl, a chelator with a strong preference for iron. As in the previous experiments, we normalized growth in dipyridyl-treated 7H9 to total growth in standard 7H9 media. Like in the iron-depleted chelexed media, both the H37Rv and Erdman ∆*rip1* strains were more sensitive to dipyridyl than the WT or complemented strains (Fig. 1G). Furthermore, this effect was observed in agitated cultures, demonstrating the effect was not due to static growth in 96-well plates (Fig. 1H). To demonstrate that this phenotype was iron dependent, we added an excess of ferric chloride, relative to the dipyridyl, and rescued the growth deficiency of the H37Rv ∆*rip1* strain (Fig. 1I).

The inability of the ∆*rip1* strain to grow in low-iron conditions might have indicated a general dysregulation of iron homeostasis in this mutant. Therefore, we tested whether this mutant was sensitive to relatively high concentrations of iron in 7H9. We found that the loss of *rip1* did not alter sensitivity to high iron concentrations up to 3 mM (Fig. 1J). Taken together with our observations using iron-depleted chelexed media and dipyridyl, we conclude that *rip1* is required for growth in iron-limited conditions.

### Rip1 is required for growth in low-zinc conditions

**Fig 2:**
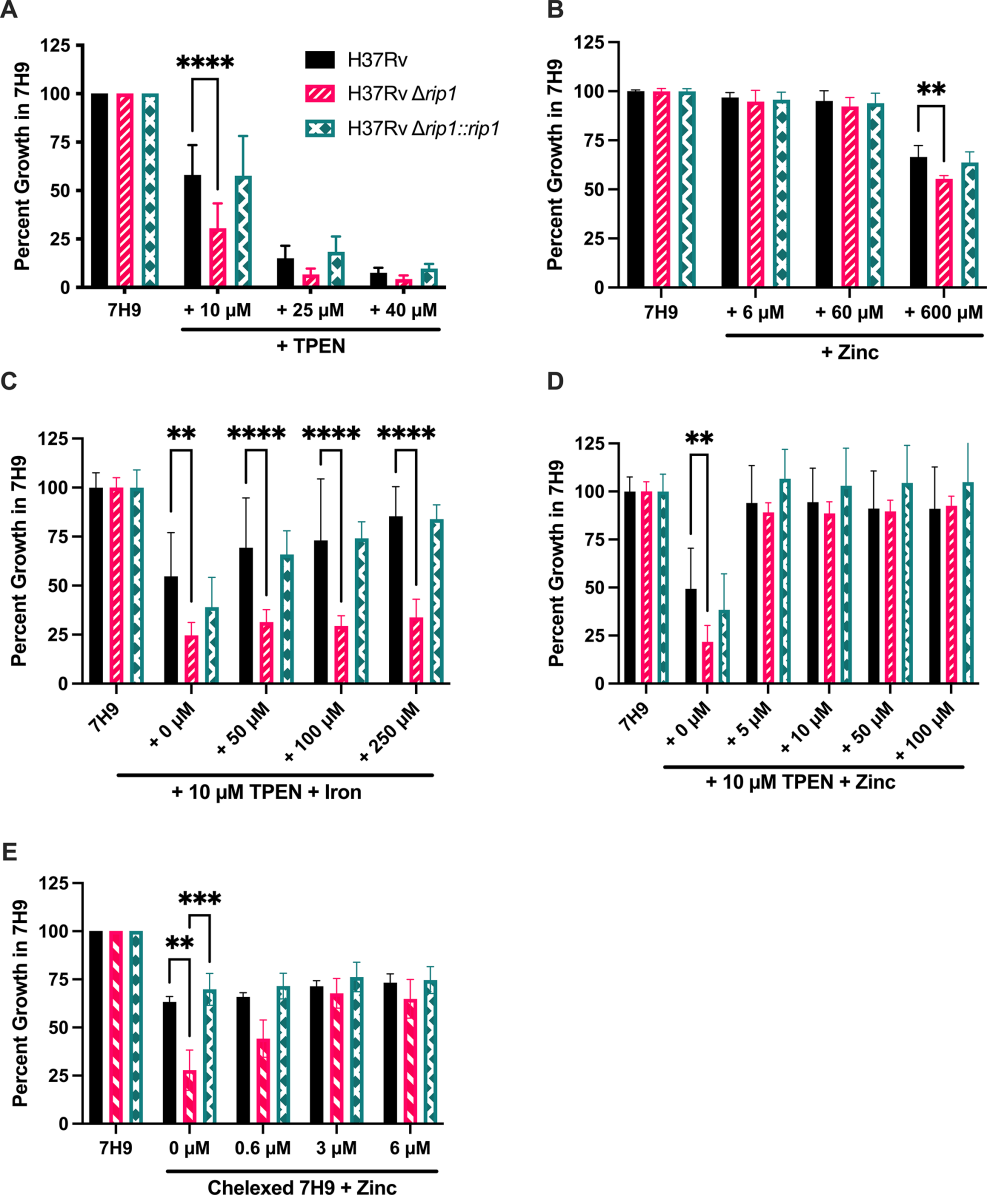
The ∆*rip1* strain is sensitive to zinc limitation but not intoxication. Growth completed in 96-well plates, incubating standing at 37° for 7 days, and measured by OD at 600 nm. Data shown are mean and standard deviation for experiments noted for each panel unless otherwise stated. (**A**) As calculated for Fig. 1, percent growth for H37Rv strains in standard 7H9 with 10 µM, 25 µM, or 40 µM TPEN. Five experiments performed in technical duplicate. (**B**) Percent growth with 6 µM, 60 µM, or 600 µM of additional zinc. Data shown are the mean and standard deviation for combined data from two experiments performed in technical duplicate. (**C and D**) Percent growth in standard 7H9 with 10 µM TPEN with additional iron (**C**) or zinc (**D**) at stated concentrations. Data shown are the mean and standard deviation for combined data from two experiments performed in technical triplicate. (**E**) Percent growth for H37Rv strains in chelexed 7H9 with stated concentrations of zinc. Three experiments in technical triplicate. Statistical analysis by two-way analysis of variance with Tukey’s multi-comparison correction. ****P < 0.0001; ***P < 0.001; **P < 0.01.

The homeostatic regulation of different metals tends to be interlinked due to common mismetallation events and, in the case of iron and zinc, overlapping transcriptional responses to starvation (12, 13, 49). As Rip1 has already been implicated in the responses to copper intoxication and restriction in Mtb, we sought to extend these studies to zinc (46, 50). Thus, we tested whether the ∆*rip1* strain was sensitive to zinc starvation using TPEN, a chelator with preferential affinity for zinc. Growth of the ∆*rip1* mutant was significantly more restricted in the presence of TPEN (Fig. 2A) than WT or the complemented mutant. As we observed for iron, deleting *rip1* did not produce sensitivity to high levels of zinc (Fig. 2B). To verify that TPEN sensitivity was due to zinc depletion and not iron depletion, we supplemented TPEN-chelated media with excess iron (Fig. 2C) or zinc (Fig. 2D). While supplementing with an equimolar amount of zinc reversed the growth inhibitory effect of TPEN, iron had no effect, even if added in 25-fold molar excess. To further confirm the inability of the ∆*rip1* mutant to grow in low-zinc conditions, we created a zinc-limited chelexed media and measured growth in defined amounts of zinc. Similar to what was observed with the TPEN-containing 7H9, the growth of the ∆*rip1* mutant was significantly inhibited compared with the WT and the complemented mutant when no zinc was added to the media (Fig. 2E). Up to 3 µM of zinc was needed for the growth of the ∆*rip1* strain to be comparable with the WT and complemented mutant. Overall, these studies confirmed that *rip1* is required for growth in either low-iron or low-zinc conditions.

### Construction of a barcoded sigma factor deletion mutant collection

Rip1 controls activation of numerous sigma factors via cleavage of their cognate anti-sigma factors (39, 42), but *M. tuberculosis* lacking Rip1 also has phenotypes, such as copper sensitivity, that are independent of known sigma factor pathways. To test whether the inability of the ∆*rip1* mutant to grow in low iron was due to one of its known sigma factor partners (SigDKLM) or a previously unappreciated sigma factor pathway, we took an unbiased approach, making individual deletion mutants lacking each sigma factor, excluding the essential *sigA* gene.

**Fig 3:**
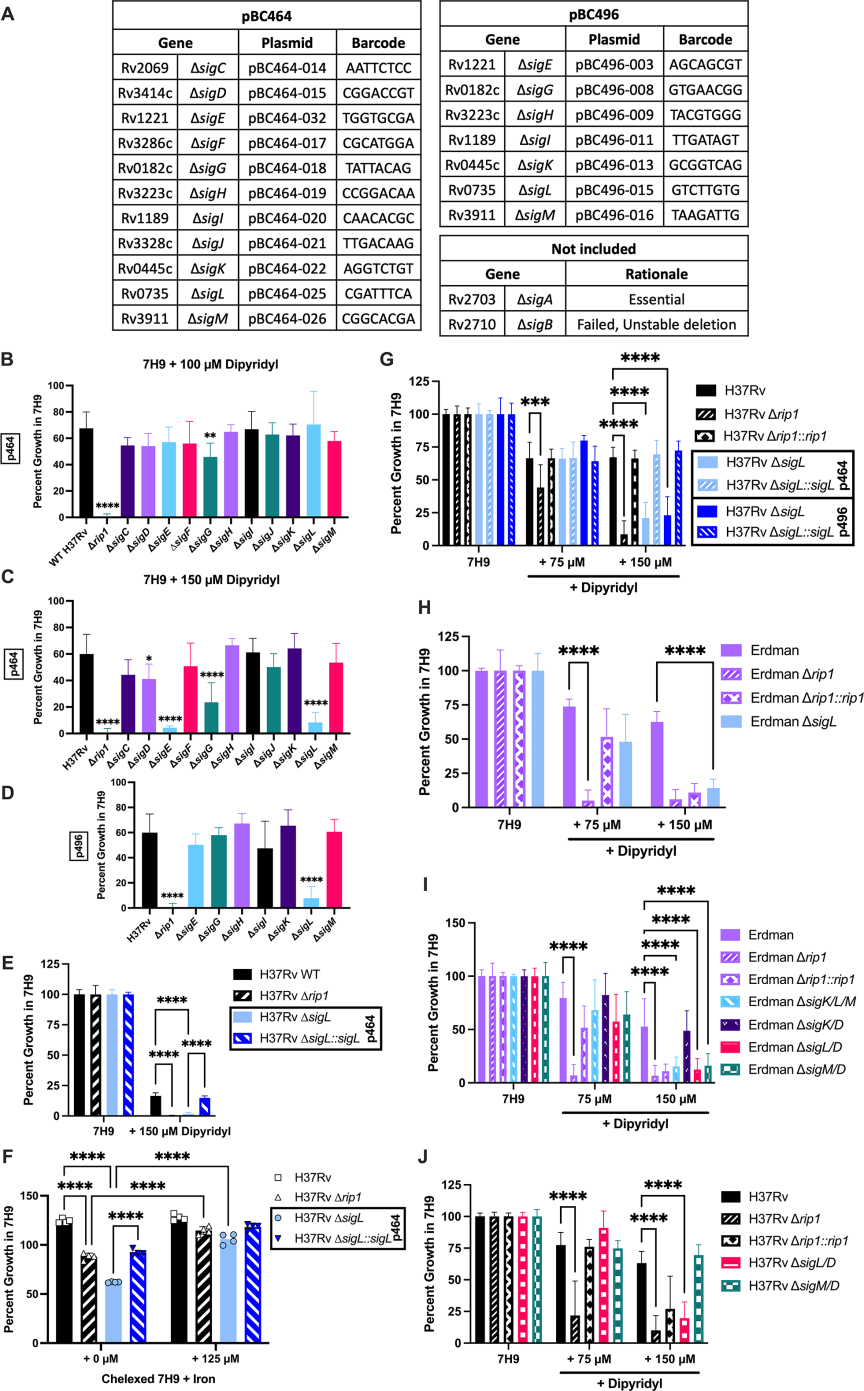
*sigL*, encoding a Rip1-regulated sigma factor, is required for growth in low iron. (**A**) Sigma factor mutant collection created in the H37Rv background for this study. Barcoded ORBIT plasmid numbers and associated barcode included for each strain. (**B–D**) Growth was measured by OD at 600 nm after 7 days of static growth in 96-well plates. Percent growth in standard 7H9 with 100 µM (**B**) or 150 µM (**C and D**) dipyridyl. Data for pBC464 (p464) strains shown in (**C**) and for pBC496 (p496) strains in (**D**). The significance of growth defect for each strain is compared with WT H37Rv. Mean and standard deviation of four experiments are shown. (**E**) Percent growth of indicated strains in agitated broth cultures in standard 7H9 or with 150 µM of dipyridyl. Mean and standard deviation shown for two experiments completed in technical triplicate. (**F**) Percent growth of indicated strains grown in 96-well plates in chelexed 7H9 media with indicated levels of added iron. Representative data shown for three experiments performed in technical quadruplicate. (**G–J**) Percent growth measured in standard 7H9 with 75 µM dipyridyl or 150 µM dipyridyl for the indicated Erdman strains (**H and J**) or H37Rv strains (**G and I**). Data depict mean and standard deviation of three (**G and H**) or five experiments (**I and J**). All experiments performed in technical duplicate in 96-well plates unless otherwise noted. Statistical analysis by two-way analysis of variance with Tukey’s multi-comparison correction. ****P < 0.0001; ***P < 0.001; **P < 0.01; *P < 0.05.

The deletion mutants were made using ORBIT to replace each sigma factor-encoding gene with a selectable plasmid. To account for polar effects on genes co-transcribed with the target sigma factors, we made the deletion mutants using two plasmids, differing by the presence of a promoter placed to drive downstream gene expression in pKM496, which is absent from pKM464. In addition, to enable the individual assessment of strain fitness in pooled experiments, we added a random 8-mer barcode into the pKM464 and pKM496 backbones to make pBC464 and pBC496, respectively. Unique barcodes were chosen for pBC464 and pBC496 to allow pooling of all strains, regardless of which plasmid was used to generate the mutant. With these plasmids, we made individual mutants, targeting each of the sigma factors in H37Rv (Fig. 3A). All the mutants were constructed with the barcoded pBC464 plasmids. The sigma factors with downstream genes in the same orientation also had deletion mutants made with pBC496 to allow for variation in the strength of downstream gene expression. The only deletion mutant that was not successfully included was ∆*sigB*, which could be deleted, but the resulting mutant was inefficiently recovered from cryopreservation. In sum, 18 individually barcoded sigma factor deletion mutants were constructed.

### SigL contributes to growth in low-iron conditions

To determine which sigma factor might be acting downstream of Rip1, each of the deletion mutants was grown in 7H9 with various levels of dipyridyl. In the presence of 100 µM dipyridyl, which ablates the growth of a ∆*rip1* mutant, the sigma factor mutants grew similarly to WT except for ∆*sigG*, which showed slightly reduced growth (Fig. 3B). Thus, none of the single sigma factors completely reproduced the ∆*rip1* phenotype. However, at 150 µM dipyridyl, the pBC464 deletion mutants of ∆*sigE*, ∆*sigG*, and ∆*sigL* were all significantly impaired in their growth compared with WT (Fig. 3C). For the pBC496 deletion mutants that support higher levels of downstream gene expression, only ∆*sigI* and ∆*sigL* were significantly affected (Fig. 3D). When all data were compared, only ∆*sigL* was consistently affected by the dipyridyl regardless of the targeting plasmid used. This observation, in addition to its known regulation by Rip1, made SigL the most compelling candidate. To confirm the role of SigL in this pathway, we measured growth in agitated cultures and demonstrated the growth of the ∆*sigL* strain was significantly inhibited in the presence of 150 µM dipyridyl (Fig. 3E). In addition, growth was significantly reduced for the ∆*sigL* strain in iron-depleted chelexed 7H9 (Fig. 3F), confirming the observed phenotype is due to iron restriction. Complementation of the ∆*sigL* strains with a second copy of the gene was sufficient to restore WT levels of growth in 150 µM dipyridyl (Fig. 3G). The connection was further strengthened by observing that the ∆*sigL* strain of Erdman possessed a similar phenotype (Fig. 3H).

The relatively severe phenotype of ∆*rip1* compared with ∆*sigL* suggested the possibility of redundancy with other sigma factors. To investigate the possibility, we took advantage of existing mutants in the Erdman background (42, 46). ∆*sigK*/∆*sigL*/∆*sigM* (∆*sigK*/*L*/*M*), ∆*sigD*/∆*sigK (*∆*sigD/K*), ∆*sigD*/∆*sigL (*∆*sigD/L*), and ∆*sigD*/∆*sigM (*∆*sigD/M*) strains were grown in dipyridyl-treated conditions as in previous experiments. Like the single sigma factor deletion mutants, none of the double or triple mutants were as susceptible to dipyridyl as the ∆*rip1* strain. At 150 µM, all the mutants were susceptible except for the ∆*sigD*/*K* strain, which grew similarly to WT (Fig. 3I). For the ∆*sigK*/*L*/*M* and ∆*sigD*/*L* mutants, this effect was likely related to the deletion of *sigL*. The inability of the ∆*sigD*/*M* strain to grow under these conditions was unexpected, as neither single mutant shares this phenotype (Fig. 3C and D). To determine if these phenotypes depended on strain background, we generated double mutants for *sigD*/*L* and *sigD*/*M* in H37Rv. Deletion of *sigD/L* produced the same phenotype in both genetic backgrounds, resulting in a growth defect in the presence of dipyridyl (Fig. 3J). In contrast, the H37Rv ∆*sigD*/*M* strain grew like WT under these conditions, suggesting the possibility of strain-dependent effects. Overall, the deletion of *sigK*/*L*/*M* in Erdman and *sigD*/*L* in either strain background did not increase sensitivity to dipyridyl compared with the ∆*sigL* mutant, indicating that SigK, SigM, and SigD do not contribute to the regulation of iron homeostasis and confirming a distinct role for SigL in this process.

### ∆*rip1* and ∆*sigL* signal iron starvation at lower levels of chelation than WT

**Fig 4:**
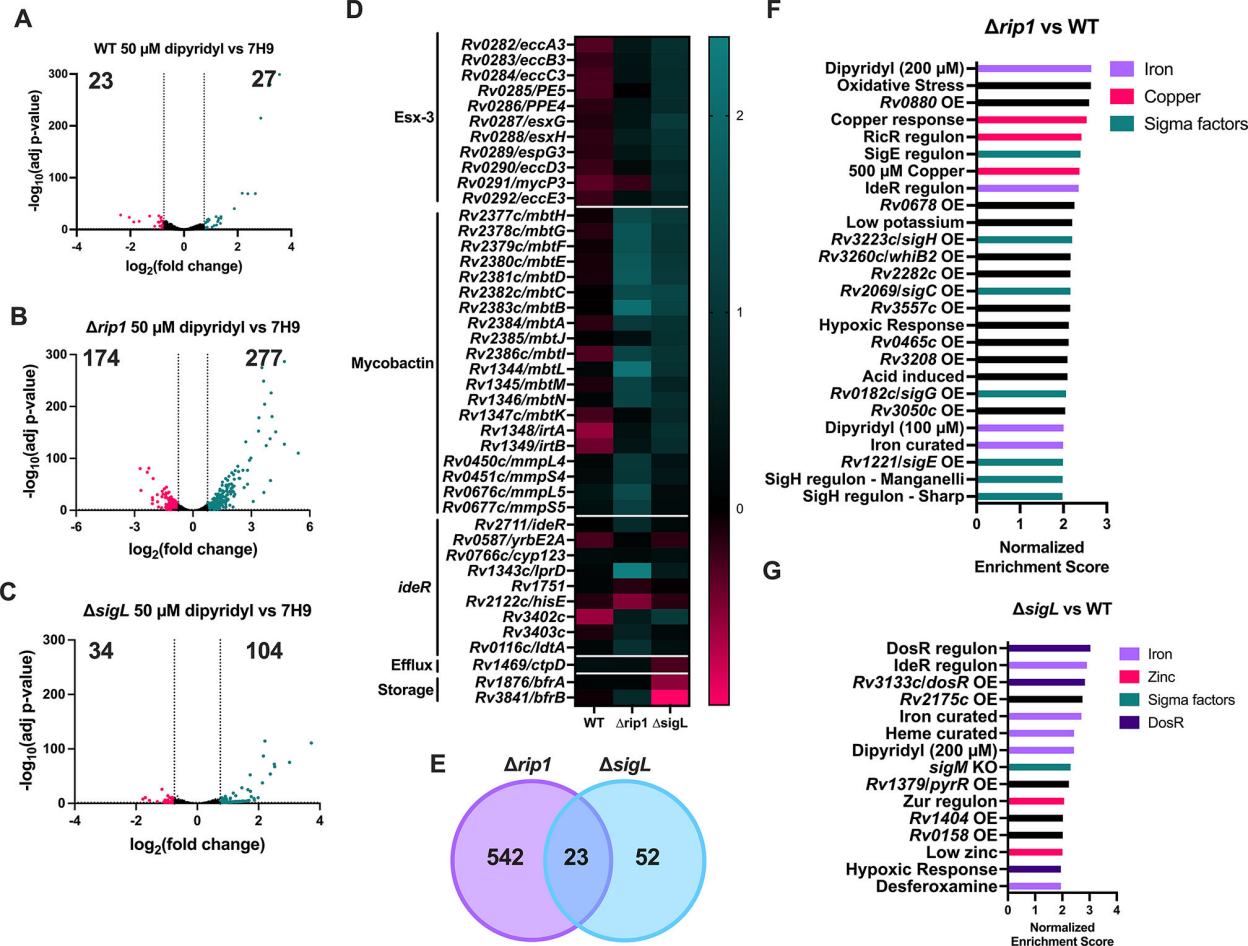
∆*rip1* and ∆*sigL* strains respond to low-iron conditions but signal iron starvation at lower levels of dipyridyl compared with WT H37Rv. Plots of log_2_ fold change vs −log_10_ adjusted *P*-value for WT (**A**), ∆*rip1* (**B**), and ∆*sigL* (**C**) comparing each strain in 50 µM dipyridyl vs standard 7H9. Significantly upregulated (teal) and downregulated (pink) genes highlighted (log_2_ fold change >0.75 and adjusted *P*-value < 0.05) with the number of genes in each category in the corresponding quadrant. Samples tested in triplicate except for ∆*rip1* in 50 µM dipyridyl which is in duplicate. (**D**) Heatmap of the log_2_ fold changes for IdeR-regulated genes for WT, ∆*rip1*, and ∆*sigL* comparing 50 µM dipyridyl to standard 7H9. (**E**) Overlap of differentially expressed genes (log_2_ fold change >0.75) in ∆*rip1* and ∆*sigL* compared with WT in 50 µM dipyridyl. (**F and G**) Gene set enrichment analysis comparing the mutants to WT in 50 µM dipyridyl treatment. Gene sets with a false discovery rate less than 0.05 were graphed with their normalized enrichment scores for ∆*rip1* (**F**) and ∆*sigL* (**G**).

To understand the previously uncharacterized roles for Rip1 and SigL in the adaptation to low-iron conditions, we investigated the regulatory relationship between Rip1, SigL, and iron homeostatic gene expression. We performed RNA-seq on WT H37Rv, ∆*rip1*, and ∆*sigL* strains in 7H9 and dipyridyl-treated 7H9. We used 50 µM dipyridyl as the treatment condition since this concentration allows normal growth of WT and ∆*sigL* strains and only modestly affects the growth of the ∆*rip1* mutant (Fig. 1G and 3G). After four doublings, the strains were harvested and processed for RNA-seq. To first assess how each strain responded to iron chelation, we compared their expression profiles in 50 µM dipyridyl to untreated 7H9. As anticipated from its normal growth rate in this concentration of dipyridyl, the transcriptional profile of WT Mtb was only minimally perturbed by this treatment. Only 50 genes were found to be differentially expressed (greater than 0.75 log_2_ fold change with an adjusted *P*-value below 0.05). In contrast, we observed a more robust response in both mutants. In the ∆*rip1* and ∆*sigL* strains, 451 and 138 genes were significantly affected, respectively (Fig. 4A through C; Table S1).

We initially investigated whether a defect in the canonical IdeR-dependent iron starvation response might be responsible for the relative inability of ∆*rip1* and ∆*sigL* mutants to grow in low-iron conditions. To do this, we specifically assessed the expression of the major systems involved in iron homeostasis such as mycobactin import (*irtAB*, Esx-3), export (*mmpL4/5*, *mmpS4/5*), and synthesis (*mbtA-M*), along with other genes in the IdeR regulon. We observed that in this concentration of dipyridyl, the IdeR regulon was induced in both mutants but not in WT (Fig. 4D). In particular, genes related to mycobactin metabolism and transport were consistently and specifically induced in both mutants. These observations indicated that the recognized transcriptional response to iron starvation remains intact in the absence of Rip1 and SigL and that the low-iron growth defect displayed by these mutants is unlikely to be related to an inability to express these genes. Instead, these data indicated that the loss of Rip1 or SigL leads to the induction of IdeR-dependent gene expression under moderately iron-limited conditions where WT is relatively unaffected.

To further investigate the basis of the mutant’s phenotypes, we compared the transcriptional profiles of each mutant with WT in the 50 µM dipyridyl condition. As expected from the known regulatory relationship between Rip1 and SigL, 30% of the 75 genes differentially expressed in the SigL mutant was also perturbed in the absence of Rip1, a statistically significant overlap (*P* = 1.8 × 10^−4^) by Fisher’s exact test (Fig. 4E; Table S2). The loss of Rip1 also uniquely affected the expression of a larger set of 542 genes, consistent with its broader regulatory role. Among these effects was the previously described differential expression of *bfrB*. mRNA for this gene was 2.46-fold more abundant in the ∆*rip1* strain compared with WT but was not significantly affected by the loss of SigL (Table S1). To better understand the functional significance of these transcriptional changes, we used GSEA (51, 52). Gene lists were generated based on curated sets of functionally annotated genes, as well as genes that are differentially expressed by Mtb under diverse conditions, in mutant strains, or in strains overexpressing known transcriptional regulators (11, 12, 14, 19, 24 - 36 - 39 - 34 - 36 - 39 - 44 - 53 - 69) (Tables S3 to S5). For both mutants, most of the highly associated gene sets were related to iron homeostasis (Fig. 4G and H; Tables S4 and S5). The only other similar gene sets associated with both mutants were copper- or zinc-related genes, which were upregulated in the ∆*rip1* or ∆*sigL* mutants, respectively. This unbiased analysis confirmed that the common feature of the two mutant strains was metal ion stress, which was primarily iron related.

Additional gene sets were associated with each mutant individually. The ∆*rip1* mutant’s expression profile was enriched for gene sets related to several sigma factors, including *sigE*, *sigH*, *sigC*, and *sigG* (Fig. 4D), none of which have been previously reported to be regulated by Rip1. ∆*rip1* also matched a gene set for the oxidative stress response. Since SigE and SigH are regulated in response to oxidative stress, this suggested that ∆*rip1* was experiencing greater oxidative stress under dipyridyl treatment compared with WT or ∆*sigL*. In contrast to the relatively broad transcriptional effects of Rip1 mutation, fewer gene sets were specifically associated with the ∆*sigL* strain. In addition, we did not observe an association with gene sets of previously defined SigL-regulated genes (28, 34). This is likely attributable to experimental design differences, as previous studies profiled expression changes in *sigL*-overexpressing strains in standard 7H9 media. Most notably, DosR and hypoxia-related genes were specifically enriched in the ∆*sigL* profile. We employed standardized processing procedures to limit gene expression changes during sample prep, and we only noted DosR regulon induction in the ∆*sigL* strain. However, we note that the DosR regulon can be rapidly induced, and this observation should be interpreted with caution. Regardless, given that iron is redox active and both oxidative stress resistance and the redox-responsive DosRST system rely on iron as a cofactor or a sensor, these strain-specific gene sets could represent different consequences of the common iron-starved phenotype experienced by both mutants (70 - 72).

## DISCUSSION

In this work, we demonstrate a new role for Rip1 in the adaptation to iron and zinc limitation. Using a collection of barcoded sigma factor mutants, we showed that the Rip1-regulated sigma factor, SigL, is also involved in iron homeostasis and required for growth during iron limitation. Despite the primary roles for both Rip1 and SigL in transcriptional regulation, we found that these phenotypes were not due to a defect in the expression of canonical iron-scavenging functions. Instead, our findings suggest that these regulatory proteins influence other aspects of iron homeostasis, which is likely important for adapting to the iron- and zinc-limiting conditions encountered during infection.

We conclude that Rip1 and SigL facilitate Mtb growth in low-iron conditions. This conclusion is based on the growth defect of mutants lacking these genes in low-iron media and the similarity between their transcriptional responses to iron chelation. However, the low-iron growth defect caused by Rip1 deletion was more severe than that caused by loss of SigL, and we were unable to associate other sigma factors with this phenotype either singly or in combination. While we cannot rule out a complex contribution from an untested combination of sigma factors, the lack of a consistent low-iron growth defect for any strain of our unbiased mutant library suggests a sigma factor-independent activity of Rip1 is involved. This model is supported by the recent observation that Rip1 controls the expression of a small RNA independently of its known sigma factor targets (46). We anticipate that the barcoded sigma factor library will prove equally useful in deciphering other complex regulatory cascades.

Given that Rip1 and SigL are well-characterized regulators of promoter activity, it was somewhat surprising that the low-iron growth defects observed for mutants lacking these proteins was not reflected in the response of iron-regulated genes to this stress. Not only was the canonical iron-starvation response active in these mutants, it was triggered at a lower degree of chelation than WT. Similarly, we found that many genes known to be repressed by the iron-sparing response, which reduces iron requirements during starvation, were appropriately downregulated in dipyridyl treatment in the ∆*rip1* strain (Table S6), providing more evidence that the response to low iron is intact (73). These data indicate that other aspects of metal homeostasis, such as import or intracellular sequestration, may be responsible for the observed inability to adapt to low-iron conditions. A role for sequestration is suggested by the increased expression of the iron-storage gene, *bfrB*, that is caused by *rip1* deletion. However, *sigL* deletion did not affect *bfrB* expression. Thus, this mechanism would be specific to *rip1* deficiency and may only account for the relatively increased iron requirement of this mutant, relative to the ∆*sigL* strain. Alterations in the cell envelope that inhibits iron import could explain the common defects in both mutants. The loss of Rip1 leads to changes in cell envelope lipids, and SigL regulates approximately 20 genes related to the cell envelope and secreted proteins, though we did not observe correlated changes in their expression in our data (28, 34, 48).

This work contributes to our understanding of the complex role of Rip1 during infection. Similar to our findings under low-iron conditions, the dramatic fitness defect of Rip1-deficient mutants in the mouse lung is not reproduced by mutation of its known sigma factor targets (28, 42, 44, 46, 53). Instead, this defect was recently reported to be related to the sigma factor-independent expression of a copper-chelating chalkophore that imparts resistance to nitric oxide (46). However, mutation of the inducible nitric oxide synthase in the mouse only partially relieved the fitness defect of Rip1-deficient Mtb, demonstrating that this mutant is sensitive to additional host-derived stresses, such as the iron and zinc starvation sensitivities we describe. Together, these findings suggest that Rip1 contributes to Mtb growth and survival during infection by coordinating several aspects of metal homeostasis via a combination of sigma factor-dependent and -independent mechanisms.

## MATERIALS AND METHODS

### Culture and growth assays

All strains were cultured in standard 7H9 (4.7 g/L Middlebrook 7H9 powder, 10% vol/vol oleic acid, albumin, dextrose, and catalase, 0.05% vol/vol Tween 80, and 0.2% vol/vol glycerol), unless otherwise stated. Zeocin (25 µg/mL), hygromycin (50 µg/mL), or kanamycin (25 µg/mL) were added as necessary. For chelexed 7H9 media, 4.7 g/L Middlebrook 7H9 powder with 0.2% dextrose, 0.05% Tween 80, and 0.2% glycerol was stirred with 10 g/L chelex overnight. Media was filter sterilized, and the following metals were added from filter-sterilized stocks: copper sulfate to 6.3 µM, magnesium sulfate to 415.4 µM, and calcium chloride to 4.5 µM. For iron-limited media, zinc sulfate was added to 6.2 µM, and iron was supplemented at stated concentrations using filter-sterilized ferric chloride in water. For zinc-limited media, ferric chloride was added to a final concentration of 250 µM, and zinc was supplemented at stated concentrations using filter-sterilized zinc sulfate in water. Dipyridyl or *N,N,N´,N*´-tetrakis(2-pyridinylmethyl)-1,2-ethanediamine (TPEN) was dissolved in dimethylsulfoxide at a concentration of 100 mM or 50 mM, respectively, and added to standard 7H9 at specified concentrations 16 hours before use. For growth experiments, fresh cultures were started from freezer stocks, grown up to stationary phase, and reinoculated into fresh standard 7H9 for 5 days to grow to an optical density (OD) of 1. Cultures were washed three times in 1× PBS with 0.05% Tween 80. Growth curves were started at an OD of 0.05 and incubated standing at 37° for 96-well plates or in a shaking incubator at 37° for agitated cultures for 7 days. Growth was quantified by measuring OD at 600 nm. Percent growth was calculated from day 7 readings as the average OD of a strain in the specified condition divided by the average OD of the strain in standard 7H9, multiplied by 100.

### Construction of plasmids

To add 8-mer barcodes to pKM464 and pKM496 (47) to make pBC464 and pBC496, respectively, each plasmid was amplified excluding the antibiotic resistance gene with a 5′ primer including eight random nucleotides. Plasmids were reconstructed by Gibson assembly with a zeocin antibiotic resistance gene, followed by sequencing to confirm correct recombination. After transformation into DH5α, individual colonies were grown up and isolated for sequencing of the barcode. Plasmids with barcodes with a Hamming distance of at least 3 were chosen for use in oligonucleotide-mediated recombineering followed by Bxb1 integrase targeting (ORBIT) transformations. Complement plasmids were constructed using Gateway cloning followed by sequencing to confirm correct insertion of target genes.

### Construction of deletion mutants

Erdman deletion mutant strains were constructed as described previously (39, 46). Deletion mutants were made using ORBIT as described previously (47). WT H37Rv containing pKM461 was grown to an OD of 0.5, and expression of Bxb1 integrase and RecT was induced with 500 ng/mL anhydrotetracycline. Samples were treated with 3 mL of 2M glycine 8 hours following induction. After 16 hours, cells were collected by centrifugation, washed two times in 10% glycerol, and resuspended in 10% glycerol with 10% vol of original culture volume. An amount of 400 µL of cells was electroporated with 400 ng of pBC464 or pBC496 (Fig. 3) and 1 µg of targeting oligo. Samples were grown overnight in 7H9 and then selected on zeocin-containing 7H10 for 3 weeks, followed by outgrowth in zeocin-containing 7H9. Deletion of target genes verified by amplification of 5′ and 3′ junctions of the plasmid insertion site as well as loss of the targeted gene.

### RNA sequencing

Frozen stocks of the indicated strains were expanded in 7H9 for 5 days, then washed three times in 1× PBS, 0.05% Tween 80 before use in assay. Strains were inoculated to a starting OD of 0.1 in 50 mL of standard 7H9 media with 50 µM dipyridyl. Cells were grown shaking at 200 rpm at 37°C until four doublings had occurred. Cells were harvested by centrifugation at 4,000 rpm for 10 minutes, decantation of the supernatant, and resuspension in 1 mL of Trizol. Samples were lysed by bead beating at 6.5 m/s for 45 seconds in an MP Biomedicals FastPrep, three times total, resting on ice in between followed by centrifugation at 10,000 rpm for 10 minutes. Supernatant was removed to a fresh tube and phase separation with chloroform performed. RNA was purified using the Zymo Direct-zol kit. DNA was removed using NEB DNase I followed by cleanup with the Zymo clean and concentrator kit. rRNA was depleted using Illumina Ribo-Zero Plus kit. Libraries were prepped using the NEB Ultra II Directional RNA Library Prep Kit for Illumina and sequenced by 150 bp paired end reads on an Illumina HiSeq 4000. Reads were mapped to the *M. tuberculosis* H37Rv genome (Genbank accession NC_000962.3) using Burrows–Wheeler Alignment (74). Read count normalization and statistical analysis of differentially expressed genes (DEGs) were calculated using DESeq2 (75). Significant DEGs were taken to be those with an adjusted *P*-value < 0.05, after correction for multiple testing. Data are available from GEO at Accession number GSE229446 and in Table S1. Fisher’s exact test completed using GraphPad Prism 9.

### Gene set enrichment analysis

Gene sets were compiled from published data to cover a wide range of regulons and transcriptional responses to stress. Information on the gene sets and source publications is in Table S3. Gene set enrichment analysis (GSEA) was completed as published previously using the GSEA desktop application (51, 52). Briefly, analysis was completed on normalized count data from the RNA-seq with default parameters using the gene set permutation type without the collapse of gene symbols. Gene sets with a false discovery rate q-value of < 0.05 were considered significantly associated with the expression profile.

## SUPPLEMENTAL MATERIAL

Table S1 msphere.00389-22-s0001.xlsx RNA-seq data for WT, Δ*rip1*, and Δ*sigL* in 50 μM dipyridyl and standard 7H9.

Table S2 msphere.00389-22-s0002.xlsx Genes differentially expressed in Δ*rip1* and Δ*sigL* included in Venn diagram in Fig. 4.

Table S3 msphere.00389-22-s0003.xlsx Gene sets curated for Gene Set Enrichment Analysis.

Table S4 msphere.00389-22-s0004.xlsx RNA-seq data for Δ*rip1* for gene sets highlighted in Fig. 4.

Table S5 msphere.00389-22-s0005.xlsx RNA-seq data for Δ*sigL* for gene sets highlighted in Fig. 4.

Table S6 msphere.00389-22-s0006.xlsx RNA-seq data for MrsI and Iron sparing response regulated genes.

ASM does not own the copyrights to Supplemental Material that may be linked to, or accessed through, an article. The authors have granted ASM a non-exclusive, world-wide license to publish the Supplemental Material files. Please contact the corresponding author directly for reuse.
